# Analysis of segmentation ontology reveals the similarities and differences in connectivity onto L2/3 neurons in mouse V1

**DOI:** 10.1038/s41598-021-82353-7

**Published:** 2021-03-02

**Authors:** Alexander P. Y. Brown, Lee Cossell, Molly Strom, Adam L. Tyson, Mateo Vélez-Fort, Troy W. Margrie

**Affiliations:** Sainsbury Wellcome Centre for Neural Circuits and Behaviour, University College London, 25 Howland Street, London, W1T 4JG UK

**Keywords:** Neuroscience, Cellular neuroscience, Neural circuits, Visual system

## Abstract

Quantitatively comparing brain-wide connectivity of different types of neuron is of vital importance in understanding the function of the mammalian cortex. Here we have designed an analytical approach to examine and compare datasets from hierarchical segmentation ontologies, and applied it to long-range presynaptic connectivity onto excitatory and inhibitory neurons, mainly located in layer 2/3 (L2/3), of mouse primary visual cortex (V1). We find that the origins of long-range connections onto these two general cell classes—as well as their proportions—are quite similar, in contrast to the inputs on to a cell type in L6. These anatomical data suggest that distal inputs received by the general excitatory and inhibitory classes of neuron in L2/3 overlap considerably.

## Introduction

A quantitative characterization of inter- and intra-region connectivity is crucial in order to elucidate general principles and region-specific features of circuit structure and function. In the neocortex, information is processed by local networks of excitatory glutamatergic principal neurons and inhibitory GABAergic interneurons^1^. The local circuit connectivity and response properties of excitatory and inhibitory neurons have been thoroughly studied in L2/3 of rodent V1^2–13^. For instance, although GABAergic inhibitory neurons make up only ~ 20% of the cortical population, connections between excitatory and inhibitory neurons are much more dense^8,14^ than connections between excitatory neurons^3^. Moreover, local connectivity shows a high degree of specificity between functionally-related neurons^6,7,9–13^. However, despite this knowledge of connectivity at a local scale, fewer studies have examined the specificity and pattern of brain-wide, long-range input onto specific cell types in V1, and the majority of those have looked at deeper layers 5 and 6^15–17^.

Retrograde modified-rabies-virus (RV) tracing, which permits identification of monosynaptically coupled presynaptic cells, has been particularly useful for mapping long-range connectivity^18,19^. Since the modified virus is intrinsically replication incompetent, presynaptic labelling is limited to neurons directly connected to the initial rabies-infected cells. Using Cre-driver lines for population tracing^20^ from molecularly defined cell types across different layers, previous RV tracing experiments have indicated V1 directly receives a variety of non-visual, multimodal sensory and spatial information^10,15–17,21^. Further, single-cell and population tracing experiments from physiologically characterized neurons show that different functional classes of cells have partially non-overlapping input profiles^15–17^. Therefore, the anatomical origin of input onto deep layers of V1 is widespread and relates to the functional properties of postsynaptic neurons, but the extent to which these connectivity profiles differ according to specific target excitatory and inhibitory pathways in L2/3 is not known.

Given the differences in response properties, local circuit connectivity and molecular markers, a corresponding difference in the brain-wide input profile to excitatory and inhibitory cells in the cortex may also be expected. Therefore, here we have examined the extent to which long-range input profiles to glutamatergic and GABAergic populations, mainly located in L2/3, differ. We developed an analytical approach, designed specifically for hierarchical segmentation ontologies, to directly compare these profiles in individual brains. Using this approach, we demonstrate that excitatory and inhibitory cells in upper layers receive inputs from the same brain regions, and in similar proportions. As a comparison, we show that the input maps onto different cell classes in L2/3 are quantitatively more similar to one another than to input maps to a class of principal cells in L6.

## Results

**Figure 1:**
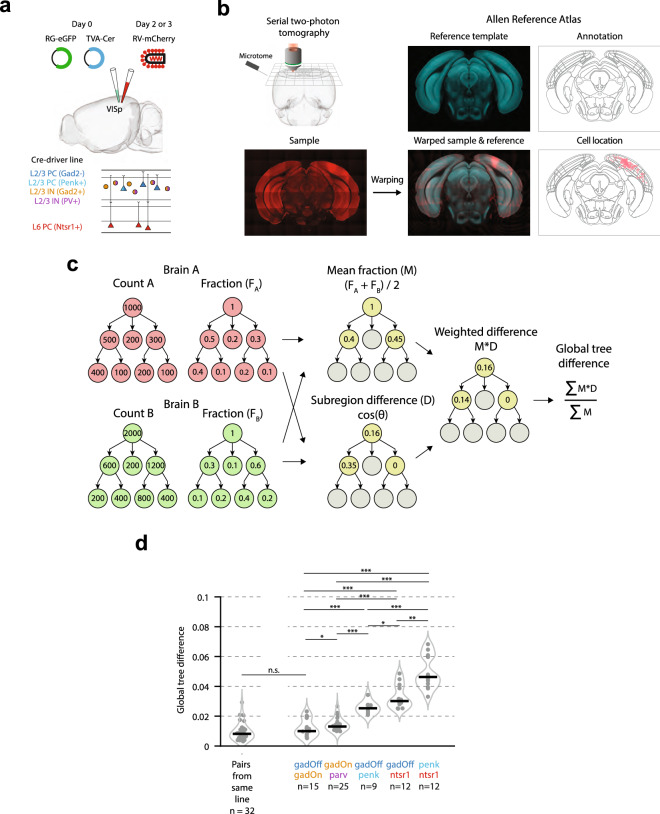
Global tree difference showing similarity of input to L2/3 relative to L6. (**a**) Schematic of experimental procedure. (**b**) Serial two-photon tomography was performed on each brain, and samples were aligned to and segmented according to the Allen Reference Atlas ontology^25^. (**c**) Outline of the algorithm used for calculating the GTD. For each brain, the cell counts in each region are converted to a fraction of the total labelled inputs. For a pair of brains, for each region, the mean fraction, M, is then calculated, as well as the cosine difference (D) between the distribution of cells within the region’s subregions. Regions with only a single subregion are excluded from further processing. Next, for each region, the mean fraction is multiplied by the cosine difference, to give a weighted difference score (M*D). Finally, this weighted difference score is summed across all regions in the hierarchy and normalized by the sum of the mean fractions across all regions. This example is intended only to demonstrate the method described, and does not reflect the data in the present study. (**d**) Global tree difference for each pair of brains from within the same line (left), and for pairs of brains between (from left to right) gadOff and gadOn, gadOn and parv, gadOff and penk, gadOff and ntsr1, and penk and ntsr1.

In order to characterise presynaptic input onto excitatory and inhibitory neurons in primary visual cortex (VISp), we used modified rabies-virus (RV) tracing^18,20^. To specifically target initial transfection to GABAergic cells in L2/3 we injected Cre-dependent AAV superficially (~ 120 μm below the pia) in Gad2-Cre and Parv-Cre transgenic mice (referred to throughout as “gadOn” and “parv”, respectively) (Fig. 1a). To target L2/3 glutamatergic, excitatory cells, we instead used a Cre-Off AAV in Gad2-Cre mice (“gadOff”)^22^, so that the AAV was selectively expressed in Gad2-negative, putative excitatory neurons. We also used superficial injections in Penk-Cre mice (“penk”), in which Cre is expressed sparsely in L2/3 excitatory neurons, as well as in a subset of L6 excitatory neurons^23,24^. In all animals, this was followed 2–5 days later by injection with a Cre-dependent rabies virus (see “Methods”;^20^) to result in line-specific monosynaptic tracing of brainwide neuronal inputs. We compared these datasets to a reanalysed dataset, which was generated by injecting the Cre-dependent AAV in Ntsr1-Cre transgenic mice (“ntsr1”)^15^, in which a subset of L6 excitatory cells were labelled.

To ensure that initially transfected neurons in gadOn and gadOff mice were targeted to inhibitory and excitatory populations, respectively, we used transgenic mice in which a tdTomato reporter was co-expressed in Gad2-Cre-positive cells, and injected either the Cre-On AAV or Cre-Off AAV (both expressing GFP). We found that in 2 mice with the Cre-Off AAV, 24/718 (3.3%) and 20/599 (3.3%) of GFP-positive cells also expressed tdTomato. Additionally, we also immunostained for GABA, and found 4/718 (0.6%) and 3/599 (0.5%) GFP-positive cells were also immunostained for GABA, suggesting that the Cre-Off virus was well restricted to Gad2-negative cells. In a further 3 mice, using the Cre-On AAV, 522/529 (98.7%), 262/265 (98.9%) and 206/208 (99.0%) of GFP-positive cells also expressed tdTomato, again suggesting that the Cre-On AAV expression was well restricted to Gad2-Cre-positive cells. These results suggest there is only a small amount of overlap of the two populations targeted with the Cre-On and Cre-Off viruses.

Following ex vivo imaging using serial two-photon tomography and cell counting, each brain was segmented, and presynaptic cells were assigned to brain regions using aMAP^25^ with the Allen Reference Atlas ontology (Fig. 1b). In total, data were acquired from 20 brains (gadOn: n = 5; parv: n = 5; gadOff: n = 3 mice; penk: n = 3; ntsr1: n = 4), and the number of labelled presynaptic cells ranged widely across experiments (gadOn: range 691–7217; parv: 993–16,944; gadOff: 420–2296; penk: 943–3947; ntsr1: 1112–6266). As the majority of labelled cells were ipsilateral to the injection site in all lines (percentage ipsilateral inputs: gadOn: 97.1 ± 2.1%; parv: 99.3 ± 0.2%; gadOff: 99.8 ± 0.1%; penk: 99.5 ± 0.3%; ntsr1: 98.0 ± 0.4%; mean ± SEM; Supplementary Fig. S1), we restricted further analysis to the ipsilateral hemisphere.

We first sought to explore the differences in input connectivity onto excitatory and inhibitory cells in L2/3. Our approach allows for precise localisation of cell somata and segmentation into brain regions to a high degree of accuracy^25^. However, the detailed analysis of brain-wide long-range connectivity data entails a number of distinct and largely unexplored analytical challenges. Firstly, the hierarchical nature of segmentation ontologies necessitates difficult and often arbitrary selection of regions for analysis. There may well be no clear criteria on which to decide at which level of granularity to analyse the data—whether to assign cells to the finest level of detail possible in the particular classification, or whether to agglomerate small subregions in to a single parent region. Secondly, the number of brain regions in modern ontologies makes addressing the problem of multiple comparisons critical in any detailed analysis of segmentations.

We therefore designed our analyses to address these concerns. First, in order to directly compare the distribution of cells amongst brain regions in two individual brains—such as presynaptic connectivity maps—we developed a pairwise scalar difference measure (the “global tree difference”; GTD) (Fig. 1c; Supplementary Figs. S2 and S3) which utilizes the entire hierarchy of an ontology. For each segmentation in a given pair, the fraction of total presynaptic input was calculated in each region of the hierarchy (Fig. 1c, left). Each brain region was then assigned a cosine distance score based on the difference in the distribution of cells amongst its child subregions in the two brains (the subregion difference, D) (Fig. 1c, middle). Next, an average fractional connectivity map was calculated for the two brains (the mean fraction, M) (Fig. 1c, middle), and used to calculate a weighted cosine distance. These calculations were summed and normalized to give a scalar measure of the difference between two individual brains (the GTD) (Fig. 1c, right).

If long-range connectivity is cell-type dependent, the profile of presynaptic labelling in experiments with the same population of starter cells should be more similar than experiment pairs with starter cell populations of different cell types. Thus, in order to establish a lower bound for the GTD, the value was first computed for all pairs of brains from within the same experimental condition (median GTD = 0.008, IQR = 0.006; n = 32 pairs; Fig. 1d, left). Next, in order to directly compare the pattern of input connectivity onto excitatory and inhibitory cells in L2/3, we computed the GTD for all pairs of brains in the gadOff and gadOn experiments (median GTD = 0.010, IQR = 0.010, n = 15) (Fig. 1d, right), and this value was not significantly different to the GTD value from all pairs within the same experimental condition (p = 0.1; Wilcoxon rank-sum test; see also Supplementary Fig. S4).

We next examined all pairs from the gadOn and parv experiments and found a significantly higher GTD value (median GTD = 0.013, IQR = 0.003, n = 25; p = 0.03), suggesting that presynaptic connectivity maps from the gadOff and gadOn lines are more similar than those from gadOn and parv. Since parv is a major subpopulation (around 40%) of all inhibitory neurons^26^, from a statistical point of view it is therefore surprising that this comparison is more different than between gadOff and gadOn. This difference was, at least in part, explained by differences in subregions of the RSP between gadOn and parv brains (RSPv and RSPagl; see “Results” below), and after removal of this region from the ontology, there was no longer a significant difference in the GTD value of the gadOff/gadOn and gadOn/parv groups (with RSP removed from ontology: gadOff/gadOn median GTD = 0.010, IQR = 0.007, n = 15; vs. gadOn/parv median GTD = 0.012, IQR = 0.003, n = 25; p = 0.12; Supplementary Fig. S5). This may demonstrate that parvalbumin-positive interneurons have a different input pattern to other interneurons, whilst at the same time highlighting how similar long-range input is to the general excitatory and inhibitory cell classes in L2/3 V1.

We further examined brains from the penk line, in which Cre is expressed sparsely in a subset of L2/3 and L6 excitatory neurons. Superficial injections of AAV and RV ensured that the majority of hosts were isolated to L2/3 (mean fraction of hosts ± s.e.m in L2/3: 86.4 ± 6.9%; n = 3 mice). The penk brains also showed differences to the other lines (Fig. 1d), including the gadOff line (gadOff/penk median GTD = 0.025, IQR = 0.005, n = 9). As the penk-expressing cells form a subset of all excitatory cells, this suggests that there are likely differences in input connectivity to genetically distinct excitatory cell classes, even within a single layer.

Given the high similarity of the gadOff and gadOn lines, we asked whether we could detect differences in input to cell types in L2/3 and to cells in other V1 layers. We therefore examined the difference between the presynaptic connectivity maps of the excitatory L2/3 gadOff brains and a subset of excitatory L6 cells (ntsr1-positive). This existing dataset^15^ was chosen to contextualise the apparent paucity of difference within L2/3 by comparison to an interlaminar analysis. Indeed, we found significantly larger GTD values between the gadOff and ntsr1 experiments (median = 0.030, IQR = 0.013, n = 12) (Fig. 1d, right), than either the pairs from gadOff and gadOn experiments (p = 1.3 × 10^–5^; rank-sum test), the pairs from gadOn and parv experiments (p = 2.0 × 10^–6^) and even the pairs from gadOff and penk (p = 0.02). Furthermore, ntsr1 and penk had the highest GTD of any of the groups (penk/ntsr1 median GTD = 0.05, IQR = 0.02, n = 12), showing that, even though the penk line showed differences to gadOff, input connectivity to excitatory and inhibitory cell types in L2/3 is relatively similar when compared to the input to an excitatory cell class in L6.

We next sought to examine whether differences in input connectivity to different V1 cell types existed, using a finer-grained analytical approach. We analysed the same five groups as for the GTD analysis (gadOff vs. gadOn; gadOn vs. parv; gadOff vs. penk; gadOff vs. ntsr1; and penk vs. ntsr1). Firstly, for each pair, the hierarchical segmentation ontology was simplified by excluding regions with no, or very few, presynaptic neurons according to prespecified criteria (Supplementary Fig. S6; see “Methods”). Next, by traversing the resulting ontology from top to bottom, each brain region comprising at least two subregions in which cells were reliably detected was studied. Rather than perform multiple statistical comparisons on each of these brain regions, which may be susceptible to false-positives, we first applied a vector method to determine whether the overall distribution of inputs among its child subregions differed between target cell types (Supplementary Fig. S7; see “Methods”). If this difference was significant, each child subregion was then examined, using a t-test to determine whether the fraction of cells in the child subregion was different between the two target cell types. Importantly, at each step, the fraction of cells in the child subregion was expressed as a fraction of total cells in the parent region, rather than the overall total number of labelled cells across the brain, eliminating positive dependence in the hierarchy.

**Figure 2:**
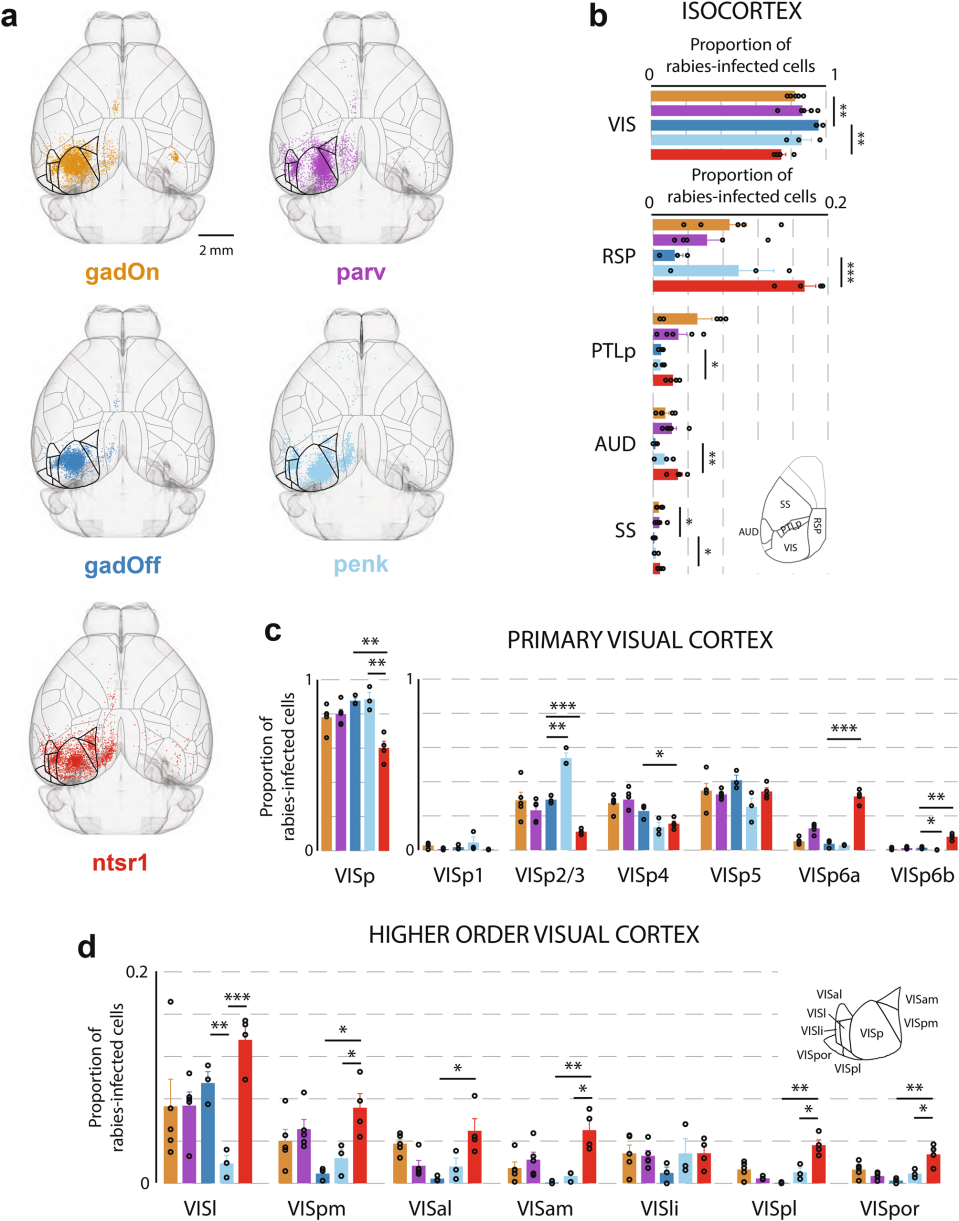
Distribution of presynaptic input from isocortex to primary visual cortex (VISp). (**a**) Horizontal projections of a template brain, showing the positions, after registration, of presynaptically connected cells within the isocortex, for each mouse line. The number of cells displayed for each line is the same, and has been normalized to the line with the fewest inputs (gadOff) by randomly sampling from all isocortex inputs. Both contra- and ipsilateral inputs are displayed, although only ipsilateral inputs were used for analysis. Horizontal surface projection of the segmentation is shown. Areas comprising visual cortex are highlighted with thicker outlines. (**b**) Proportion of labelled rabies-infected cells in ipsilateral top-level areas of the isocortex, expressed as a fraction of all cells in isocortex. Inset, location of RSP, PTLp, AUD and SS on the horizonal surface projection of the segmentation. (**c**) Left, proportions of labelled cells in VISp (expressed as a fraction of all cells in visual cortex); right, proportions of labelled cells in different layers of VISp (expressed as a fraction of all cells in VISp). (**d**) Proportions of labelled cells in subregions of the visual cortex (higher visual areas), expressed as a fraction of all cells in visual cortex. Inset, location of each visual area in the horizontal segmentation. Error bars show the S.E.M. and data from individual mice are shown as dots overlaying the bars.

As suggested by the GTD analysis above, and despite experimental groups showing significant input from many brain regions (Supplementary Fig. S8), the overall input to the gadOff and gadOn groups was highly similar, with only two regions in the entire ontology showing significant differences in fractional input connectivity: visual cortex (VIS) and somatosensory cortex (SS) (Fig. 2a,b). Within the isocortex, the gadOff line received a greater fraction of input from the visual cortex (including primary and higher areas) than inhibitory gadOn cells (gadOff: 95.7 ± 1.3%; gadOn: 82.3 ± 1.6%; p = 0.001), and a lower fraction from SS (gadOff: 0.14 ± 0.07%; gadOn: 0.66 ± 0.15%; p = 0.04). However, no other significant differences were found in the hierarchy, either cortically or subcortically. Notably, none of the particular visual cortical areas showed a difference in input fraction, expressed as a fraction of the total input from all visual areas (VIS) (Fig. 2c,d). Although locally, within the layers of VISp, differences in input fraction (expressed as a fraction of total input from VISp) were observed between the penk and the gadOff in L2/3 (gadOff: 29.5 ± 1.3%; penk: 53.8 ± 3.0%; p = 0.002) and VISp6b (gadOff: 1.39 ± 0.38%; penk: 0.17 ± 0.08%; p = 0.03), no differences were seen between the gadOff and gadOn within layers of VISp (Fig. 2c, right).

Similarly, the two L2/3 inhibitory lines (gadOff and parv) showed very few differences, with the only regions differing significantly being in the subregions of the retrosplenial cortex (RSP). Namely, a greater fraction of RSP input to parvalbumin-positive VISp neurons came from RSPagl, whereas a larger fraction of input to the superset of interneurons in the gadOn line came from RSPv.

**Figure 3:**
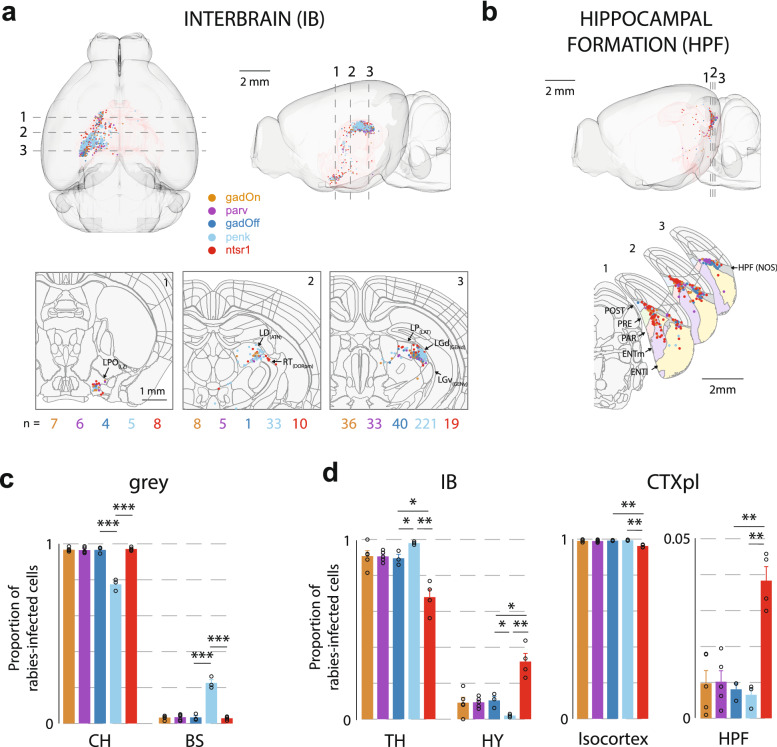
Distribution of presynaptic input from interbrain (IB) and hippocampal formation (HPF) to VISp. (**a**) Top, horizontal and sagittal projections, showing the distribution of labelled rabies-infected cells within the IB. Dashed lines indicate location of cross-section images, below. Bottom, coronal cross-sections of the template brain at three locations, with the position of labelled cells in the interbrain. Brain regions are outlined, and cells ± 20 μm anterior–posterior of the cross-section location are shown. Lateral preoptic area (LPO), lateral dorsal nucleus (LD), reticular nucleus (RT), lateral posterior nucleus (LP), and dorsal (LGd) parts of the lateral geniculate complex are indicated (in brackets, the parent regions of these regions). Numbers below indicate the number of cells from each line appearing in the image. (**b**) Sagittal projection showing the distribution of labelled cells within the HPF. Dashed lines indicate location of cross-section images, below. Below, coronal cross-sections of the template brain at three locations, with the position of labelled HPF cells and brain regions outlined (includes cells ± 10 μm anterior–posterior of the cross-section location). Pre- (PRE), post- (POST) and para- (PARA) subiculum, as well as lateral (ENTl) and medial (ENTm) entorhinal cortex are indicated. The dark grey region indicates a region labelled HPF with no further subdivision (*NOS* not otherwise specified). (**c**) Proportion of rabies-infected cells within the subregions of the interbrain (IB) and cortical plate (CTXpl). Error bars show the S.E.M. and data from individual mice are shown as dots overlaying the bars.

On the other hand, significant differences in fractional input to excitatory L2/3 cells and a subset of excitatory L6 ntsr1-positive cells were found in several regions across the brain (Figs. 2, 3). From the isocortex, L6 ntsr1-positive cells received a significantly greater fraction of input from outside of visual cortex than L2/3 gadOff cells (fraction of isocortex presynaptic inputs in VIS; gadOff: 95.7 ± 1.3%; ntsr1: 74.5 ± 2.5%; p = 0.001, t-test), including a very large fraction from RSP (gadOff: 2.5 ± 0.9%; vs. ntsr1: 17.3 ± 1.3%; p = 0.0003) (Fig. 2b). Within visual cortical regions, again L6 ntsr1-positive cells received a lower fraction of local primary visual cortex (VISp) inputs than L2/3 gadOff cells (Fig. 2c) (gadOff: 87.7 ± 1.6% of VIS inputs from VISp; vs ntsr1: 60.0 ± 3.9%; p = 0.002). Within VISp itself, there were significant differences in the distribution of inputs from different layers to gadOff and ntsr1-positive cells (including VISp2/3, VISp4, VISp6a and VISp6b; Fig. 2c, right). Indeed, in all secondary visual areas (VISpm, VISal, VISam, VISpl, VISpor) bar two (VISl and VISli) L6 received significantly more input than L2/3 gadOff cells (Fig. 2d). These data suggest that, although both L2/3 and L6 receive a wide range of inputs from similar cortical regions, L6 ntsr1-positive cells may integrate relatively more input from cortical regions outside VISp than superficial layers, which pools inputs more locally.

Outside of the isocortex no differences were observed between gadOff and gadOn, or gadOn and parv (Fig. 3). However, differences were observed between the gadOff and penk brains. Most notably a difference was observed between penk and the other lines at one of the highest levels of the ontology, grey matter (grey), with a much greater fraction of grey matter input coming from the brainstem (BS) than the cerebrum (CH) in the penk mice (fraction of grey inputs from BS; penk: 22.6 ± 1.9%; gadOff: 3.3 ± 0.5%; ntsr1: 2.9 ± 0.5% Fig. 3c). The majority of this brainstem input to penk originated in the lateral geniculate nucleus (LGd; 61.8 ± 8.3% of BS input), with smaller contributions from LP, PO, and LD (Fig. 3a; Supplementary Fig. S8), perhaps suggesting that the Penk cell class receives relatively large amounts of feedforward input from retina via LGd, relative to other cells types in L2/3.

Several regions also differed significantly in the input they provided to L6 ntsr1-positive versus L2/3 gadOff (Fig. 3). For instance, interbrain (IB) (Fig. 3a,d) inputs on to L2/3 gadOff cells were more likely to originate from thalamus (gadOff: 89.5 ± 2.3% vs. ntsr1: 67.9 ± 4.5%; p = 0.01), and, accordingly, L6 cells received significantly higher fraction of their IB input from regions of the hypothalamus (Fig. 3d). L6 ntsr1-positive cells also received a greater fraction of cortical plate inputs from the hippocampal formation (HPF) than L2/3 (gadOff: 0.81 ± 0.16% vs. ntsr1: 3.83 ± 0.39%; p = 0.002; Fig. 3b,d) including inputs from CA1 and the post-, pre- and parasubiculum, as well as lateral and medial entorhinal cortex.

## Discussion

Several recent studies in the neocortex and midbrain have examined the brain-wide distribution of direct inputs onto different cell classes (visual cortex^15–17,20^; prefrontal cortex^27,28^; somatosensory cortex^29–32^; ventral tegmental area^33,34^). Many of these studies have shown only modest effects of cell class on long-range connectivity, with all cell classes in a region generally receiving similar proportions from distant input regions. Here we have examined the distribution of whole-brain, monosynaptic input to excitatory and inhibitory neurons in L2/3 of V1 using rabies-viral tracing. Consistent with these previous studies it appears inhibitory and excitatory neurons in L2/3 of mouse V1 receive highly overlapping inputs from distal brain regions.

However, in contrast to previous studies (e.g.^27,30^) rather than use multiple Cre-lines to distinguish subpopulations of, for example, GABAergic cells, we have rather studied excitatory and inhibitory classes more generally^35^, using Cre-Off and Cre-On AAVs in Gad2-Cre mice. Thus, the primary goal of this study was not to investigate differences in innervation between the many particular subtypes of inhibitory or excitatory cells, but whether these two classes receive anatomically segregated distant inputs. For comparison, we also examined input to parvalbumin-expressing cells, the largest known subtype of GABAergic neuron in the neocortex^1^, and proenkephalin-expressing (penk) cells, a subtype of excitatory cell present in L2/3 and L6^23,24^. Although the differences were relatively small, there were noticeable qualitative trends in the fraction of inputs to the different classes, suggesting the potential for more subtle diversity in the excitatory and inhibitory populations than our study could detect.

It is clear from these data that L6 ntsr1-positive cells are readily distinguishable from upper layer cells based on the profile of their long-range input. On a global scale, and although we restricted the majority of our analysis to ipsilateral input, ntsr1-positive cells received a significantly higher fraction of contralateral inputs than gadOff, parv and penk cells, whereas none of the L2/3 lines were significantly different to each other (Supplementary Fig. S1). Further, the anatomical weighting of RSP input, which carry head-motion related signals to deep layers^36^ is substantially higher than to upper layers^15,20,37^. Indeed, ntsr1-positive cells received relatively large amounts of direct input from areas in the hippocampal formation, such as CA1^38^ and presubiculum, parasubiculum and postsubiculum^39^, which contain head direction responses^40^, and which are thought to strongly contribute to grid cell representations in entorhinal cortex^41^. Therefore, it appears processing in L6 ntsr1-positive cells utilizes information from functionally distinct long-range inputs as compared to cells in superficial layers.

Although these data indicate that both excitatory and inhibitory neurons in L2/3 receive similar information, it is important to recognize that physiological processing of that information will depend on several other key factors, such as the relative weight and dynamics of these long-range synaptic connections. Also, it will be important to determine whether individual inhibitory and excitatory cells share input from the same presynaptic cells, or rather process information in parallel, from distinct presynaptic populations.

Despite similar regions connecting in similar proportions to cell types in L2/3, different cell classes within localized portions of V1 possess distinct visual and behaviourally-relevant response properties^8,42,43^. Thus, major differences in the response profiles of these cell classes likely arise from a combination of diversity in synaptic physiology^44^, intrinsic biophysical properties of the target cells^45^ and local circuit connectivity^6,7,14^ rather than long-range, inter-regional connectivity.

Our work supplements recent studies attempting to fully describe connectivity across the mouse brain^46–48^. These labelling studies have produced large-scale, accessible connectivity datasets. However, quantitative comparison of these datasets to our data, in which retrograde tracing is restricted specifically to monosynaptic inputs onto particular cell classes, is difficult and requires certain assumptions. For example, assessing the relative connection weight from multiple source regions to a target region using anterograde tracing data^46,47^, requires normalizing for injection volume in the source regions. Thus, assumptions must be made about both the density of axonal projection per unit volume in the source region, as well as the density of synapses made by axons in the target region, both of which require further experimental evidence. Nevertheless, our data is in broad agreement with these studies, and we find large amounts of input to V1 arises from higher visual cortical areas, as well as retrosplenial, parietal, auditory, and anterior cingulate cortical areas^48^.

In summary, our data suggest that L2/3 excitatory and inhibitory populations in visual cortex receive strongly overlapping long-range inputs. These inputs are relatively local when compared to the input to ntsr1-positive cells of L6, which integrate more non-visual, multi-modal input. Thus, in upper layers within the cortical column, processing of similar long-range information in upper layers likely combines with translaminar processing to shape diverse cortical function^49,50^.

## Methods

### Transgenic animals

Conventional AAV-assisted rabies virus tracing was carried out in Gad2-IRES-Cre (interneurons; Jax 010802), Penk-IRES2-Cre (Jax 025112), PV-Cre (parvalbumin positive interneurons; Jax 008069) and Ntsr1-Cre (layer 6 cortico-thalamic cells; GenSAT 030648-UCD) mice. Gad2-Cre-tdTomato reporter mice were bred by crossing Gad2-IRES-Cre with Ai14 (Jax 007914) mice. Ntsr1 data were taken from^15^, but cell counts were reanalysed in the same manner as the other datasets. To target layer 2/3 excitatory and inhibitory neurons, we used superficial injections of Cre-inactivated or Cre-activated viral constructs in Gad2-Cre animals (here termed “gadOff’” and “gadOn”, respectively). Both male and female adult animals (P35-56) were used. All animals were bred on to a C57BL/6J background.

### Surgical procedures

All experiments were performed in accordance with the UK Home Office regulations (Animal (Scientific Procedures) Act 1986), approved by the Animal Welfare and Ethical Review Body (AWERB; Sainsbury Wellcome Centre for Neural Circuits and Behaviour) and in compliance with ARRIVE guidelines. Surgical procedures were carried out under anaesthesia, using a mix of fentanyl (0.05 mg/kg)/midazolam (5 mg/kg)/medetomidine (0.5 mg/kg). Anaesthesia was reversed at the end of the procedure using naloxone (1.2 mg/kg)/flumazenil (0.5 mg/kg)/atipamezole (2.5 mg/kg) where necessary. During all procedures, both eyes were protected by the application of ointment (Maxitrol, Alcon).

The scalp overlying the left primary visual cortex was shaved, cleaned, and the animal was placed on a warming mat controlled by an internal temperature probe to ensure core body temperature did not fall below 36 °C. After incising the scalp, a small (approx. 0.5 mm diameter) craniotomy was then drilled over the monocular portion of V1 at 0.5 mm rostral and 2.3 mm lateral to lambda using a dental drill (Osada Electric, Japan) with a 300 µm burr (Cookson). Viral injection was then performed (see below), following which the craniotomy was filled with a silicone elastomer (Kwik-Cast, WPI), and the scalp overlying closed using either sutures or cyanoacrylate glue. The animal was then recovered, using a naloxone/flumazenil/atipamezole mix, and replaced in the home cage.

### Virus injection

Viral injections were carried out using a borosilicate pipette with a long, narrow shank, with a tip broken to a diameter of around 15–20 µm. In some cases, a motorised injection system (Nanoject, Drummond) was used, however most injections were carried out using a 2 ml air-filled syringe for pressure, whilst visualising the meniscus of the viral suspension under high power magnification.

Injections in Ntsr1-Cre mice were carried out at a depth corresponding to the middle of L6 (~ 690 µm below pia). In the gadOff, gadOn, PV-Cre, and Penk-IRES2-Cre mice (targeting layer 2/3 neurons) injections were performed close to the border of layers 1 and 2 (around 120 µm below the pia), to minimise the transfection of deeper neurons. Two (out of five) gadOff mice were excluded from the analysis as they were found to contain large numbers of host neurons in layer 5 (30/66 (45%) and 10/24 (42%) of hosts in L5). In the remaining three gadOff brains, one out of nine recovered hosts was found in layer 5. No hosts could be recovered for the gadOn or parv brains, likely due to death of the initial host cells. For conventional (Cre-On) tracing in gadOn and parv mice a mix of pAAV hSyn-Flex RG-cerulean (Addgene 98221) and pAAV-EF1a-FLEX-GT (gift from Edward Callaway; Addgene 26198) was used in a 2:1 ratio. For the Cre-On penk mice, we used pAAV-EF1a-FLEX-GT and pAAV-hSyn-Flex-H2B-EGFP-P2A-N2cG (Addgene 126469). For Cre-Off tracing, a mix of pAAV-EF1a cre off EGFP-E2A-RG (Addgene 126471) and pAAV-EF1a-cre off EGFP-2A-TVA (Addgene 126470) was used. The AAV mix appropriate for the particular experiment was injected over 1–2 min in short pulses. Approximately 5–20 nL was injected for each experiment. Following the injection, the pattern of blood vessels was imaged allowing for easy identification of the injection site.

Two to five days later, rabies virus (RV, 100–150 nL) was injected at the same site, using blood vessel patterns as landmarks. Smaller volumes of AAV as compared to the RV were used, in order to cause primary infection of as many transfected neurons as possible, whilst at the same time restricting the number of host neurons so as to make cell counting practicable.

### Perfusion

Animals were perfused 10–14 days after rabies infection. Animals were deeply anaesthetized using a ketamine (200 mg/kg)/xylazine (20 mg/kg) mixture, and a blunt needle placed in the left ventricle, whilst an incision was made in the right atrium. Blood was first cleared using 100 mM phosphate buffered saline (PBS); once clear, the animal was perfused with saline containing 4% paraformaldehyde. Once fully fixed, the head was removed and the brain carefully dissected out. The brain was further fixed in 4% PFA overnight at 4 °C, and then stored in 100 mM PBS with 0.1% azide to prevent bacterial contamination, until ready for imaging.

### Imaging

For serial two-photon imaging, on the day of imaging, brains were removed from the PBS and carefully dried. They were embedded in agarose (4%) using a custom alignment mould to ensure that brain was perpendicular to the imaging axis.

After trimming, the agarose blocks were transferred to the serial two photon microscope containing an integrated vibrating microtome and motorized x–y–z stage^51,52^. Two-photon imaging was carried out using 930 nm or 800 nm illumination. Images were acquired with a 1 μm pixel size, and 5 μm plane spacing. 10 optical planes were acquired over a depth of 45 μm in total. In order to image the entire brain, mosaic scanning was employed, in a 13 × 9 grid with each tile measuring 1080 pixels square, with 5% overlap.

After imaging each mosaic tile at all ten optical planes, the sample was automatically transferred to a microtome which removed a 50 μm slice, allowing for imaging of the subsequent portions of the sample, resulting in full 3D imaging with a voxel size of 1 μm in-plane, and 5 μm axially.

In a subset of control experiments, comparing gadOn and gadOff starter populations in V1, brains were removed from PBS and sliced coronally (50 μm) using a vibratome (Microm HM 650 V). Slices were incubated in PB (0.1 M PB, 0.1 M NaCl, pH 7.4) solution containing 1% bovine serum albumin and 0.1% Triton-X for 3–5 h at room temperature. For immunostaining, antibodies against GABA and EGFP were used, slices were incubated in PB with 0.1% BSA and 0.1% Triton-X containing mouse anti-GABA primary antibodies (Sigma-Aldrich A0310; 1:1000) and chicken anti-EGFP (Life Technologies, A10262; 1:1000) at room temperature o/n. After washing in PB, goat Alexa Fluor-647 anti-mouse (Life Technologies A21237; 1:500); and goat Alexa Fluor-488 anti-chicken IgY (Life Technologies A11039; 1:500) secondary antibodies were applied for 5 h. After again washing with PB, slices were mounted in 90% glycerol, 0.5% n-propyl gallate (Sigma-Aldrich, P3130), and 20 mM tris, pH 8. Slices were then imaged using a Zeiss Axio Imager 2 microscope under 20× (4 μm separation; 10–13 imaging planes per slice). Cell counting was performed manually using Zeiss ZEN blue software.

### Data analysis

#### Segmentation and cell counting

Individual tiles were preprocessed to correct for uneven illumination, and then stitched into 2D whole-brain coronal slices using custom routines written in Python or StitchIt written in MATLAB (https://github.com/SainsburyWellcomeCentre/StitchIt). Slices were then loaded as 3D stacks and inspected using either the MaSIV package (https://github.com/SainsburyWellcomeCentre/masiv) or napari (https://github.com/napari/napari). Cells were manually counted in each brain using a cell counter plugin for MaSiV, or cellfinder_curate, part of cellfinder (version 0.4.7b0, https://github.com/brainglobe/cellfinder^53^). To aid counting in cases where perfusion was imperfect, images underwent linear unmixing using emission spectra derived from pure samples of EGFP, mCherry, and blood.

Rabies-infected cells were identified manually as smooth objects with diameter of 8–30 μm and fluorescence only in the red channel (or red and green channels, in the case of host neurons in the primary injection site). Neuronal processes were often visible, further aiding identification.

Image stacks were downsampled and aligned to the Allen Reference Atlas^54^ using the aMAP algorithm described by^25^ (available at https://github.com/SainsburyWellcomeCentre/aMAP), or brainreg described in^55^ (available https://github.com/brainglobe/brainreg), both based upon NiftyReg^56^. After registration, images were segmented according to the atlas and cells were assigned to brain regions automatically.

#### Simplification of the ontology

We used the Allen CCFv3 (Oct 2016) ontology and applied the following selection criteria of regions for further analysis. Areas containing no neurons were excluded from analysis. Additionally, regions were only included for analysis provided the following conditions were met, in at least one line: first, the region must have at least 5 neurons, on average, across all brains in a line; second, the region must have at least 1 neuron in the majority (> 50%) brains in a line. In order to analyse and compare how presynaptic cells were distributed between the subregions of a particular region, we separated it into those subregions which passed the above criteria and a “not otherwise specified” (NOS) subregion. The NOS subregion was the amalgamation of all the cells in the subregions not passing the above criteria, as well as cells which were assigned to the parent region directly (i.e. not assigned to a particular subregion). The NOS subregion was not analysed for differences between lines (t-tests below), but did contribute when computing the vector space subregion analysis (see below). This step, as well as the vector space subregion analysis and subsequent t-tests, were performed separately on five groups: inter-laminar excitatory (gadOff and ntsr1; penk and ntsr1), inter-cell-class L2/3 (gadOff and gadOn), excitatory L2/3 (gadOff and penk) and inhibitory L2/3 (gadOn and parv).

#### Vector space subregion analysis

Once simplified, each region with at least two remaining subregions (not including the NOS subregion) was first analysed in order to determine whether the fraction of cells in each subregion differed systematically by host line. This step reduces the number of subsequent t-tests required (see below).

For a given region containing *n* subregions, the fraction of cells in all subregions was expressed as an n-dimensional vector for each brain. The direction of this vector provides a complete description of the cellular subregional composition for a region in a single brain. The angle between any two such vectors defines a measure of the overall similarity of the vector directions. For data such as these, which are bounded in [0, 1] in all dimensions, the cosine of the angle between the two vectors is commonly used, varying from 0 for orthogonal vectors to 1 for vectors with identical direction.

Cosine distance was therefore calculated for all pairs of experiments from within the same line, and for all pairs of experiments from different lines. These two sets of distance measures were then compared using a one-tailed Mann–Whitney test. A significant result indicates that pairs of experiments within a line are more similar in their subregional composition than pairs of experiments between lines.

#### Difference by line of individual subregion fractions

In regions in which the vector space subregion analysis was significant (p < 0.05), we next examined each child subregion of that parent region (not including the NOS region), by using an unpaired t-test to compare the fraction of cells in each line. The distribution of cells within each region was tested using a Shapiro–Wilk test for normality. Across all lines and all regions which passed the selection criteria for that line (see above), 426/478 (89%) passed the test for normality at a significance level of 0.05.

#### Global tree difference

The Global Tree Difference was defined to be a scalar value by which a pair of segmentations can be compared (see Fig. 1c and Supplementary Figs. S2 and S3 for examples). It is defined as the average cosine distance of subregional composition for each parent region, weighted by the fractional input in the region, and normalized to the total number of cells in the two brains.

For each region in the ontology, the cosine distance between the two experiments is first calculated as outlined above. This is then weighted by the average of the fractional cell counts (the number of labelled cells in the region and all subregions, divided by the total number of labelled cells in the entire hemisphere). Lastly, the metric is normalized by dividing by the total mean fractional connectivity, to give a value bounded in [0, 1]. High values of GTD are hard to achieve, requiring orthogonal subregional composition at the highest levels of the hierarchy; therefore, in experiments in which differences are not gross, it is expected that GTD values will cluster near to 0.

## Supplementary Information

Supplementary Figures.

## Data Availability

The datasets generated and analysed during the current study are available from the corresponding author on reasonable request.
