# Supplementary material for: Analysis of segmentation ontology reveals the similarities and differences in connectivity onto L2/3 neurons in mouse V1
Source: Sci Rep. 2021 Mar 2;11:4983. doi: 10.1038/s41598-021-82353-7 (PMC7925549; doi:10.1038/s41598-021-82353-7)
Supplement: Supplementary file 1 — Supplementary Figures. [file 41598_2021_82353_MOESM1_ESM.pdf]

Analysis of segmentation ontology reveals the similarities and differences in connectivity onto L2/3 neurons in mouse V1

Supplementary Information

Alexander P.Y. Brown[1+], Lee Cossell[1+], Molly Strom[1], Adam L. Tyson[1], Mateo Vélez-Fort[1] and Troy W. Margrie[1*]

[1] Sainsbury Wellcome Centre for Neural Circuits and Behaviour, University College London, 25 Howland Street, London W1T 4JG, United Kingdom

[+] These authors contributed equally to this work

* t.margrie@ucl.ac.uk

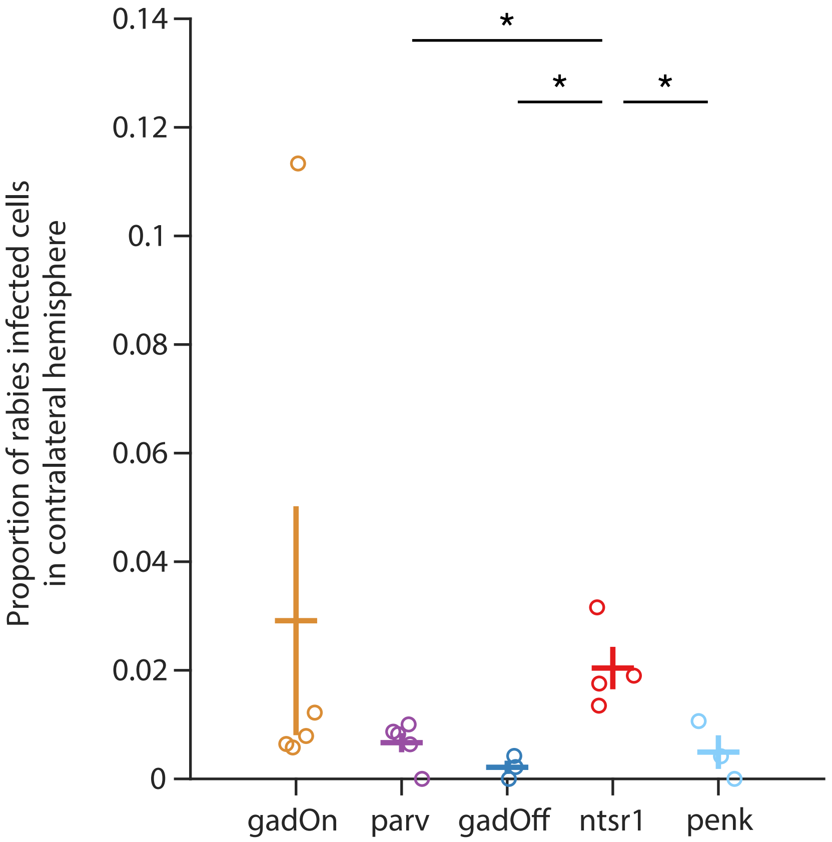

Supplementary Figure S1. Fraction of cells in the contralateral hemisphere.
Proportion of the total number of rabies-infected cells in whole brain, found in the contralateral hemisphere, for each mouse line. The proportion for each individual brain is displayed as a dot, and the mean and SEM for each mouse line are shown. The ntsr1 line had significantly more cells in contralateral hemisphere than the parv ($p = 0.011$), gadOff ($p = 0.012$) and penk lines ($p = 0.033$; unpaired t-tests).

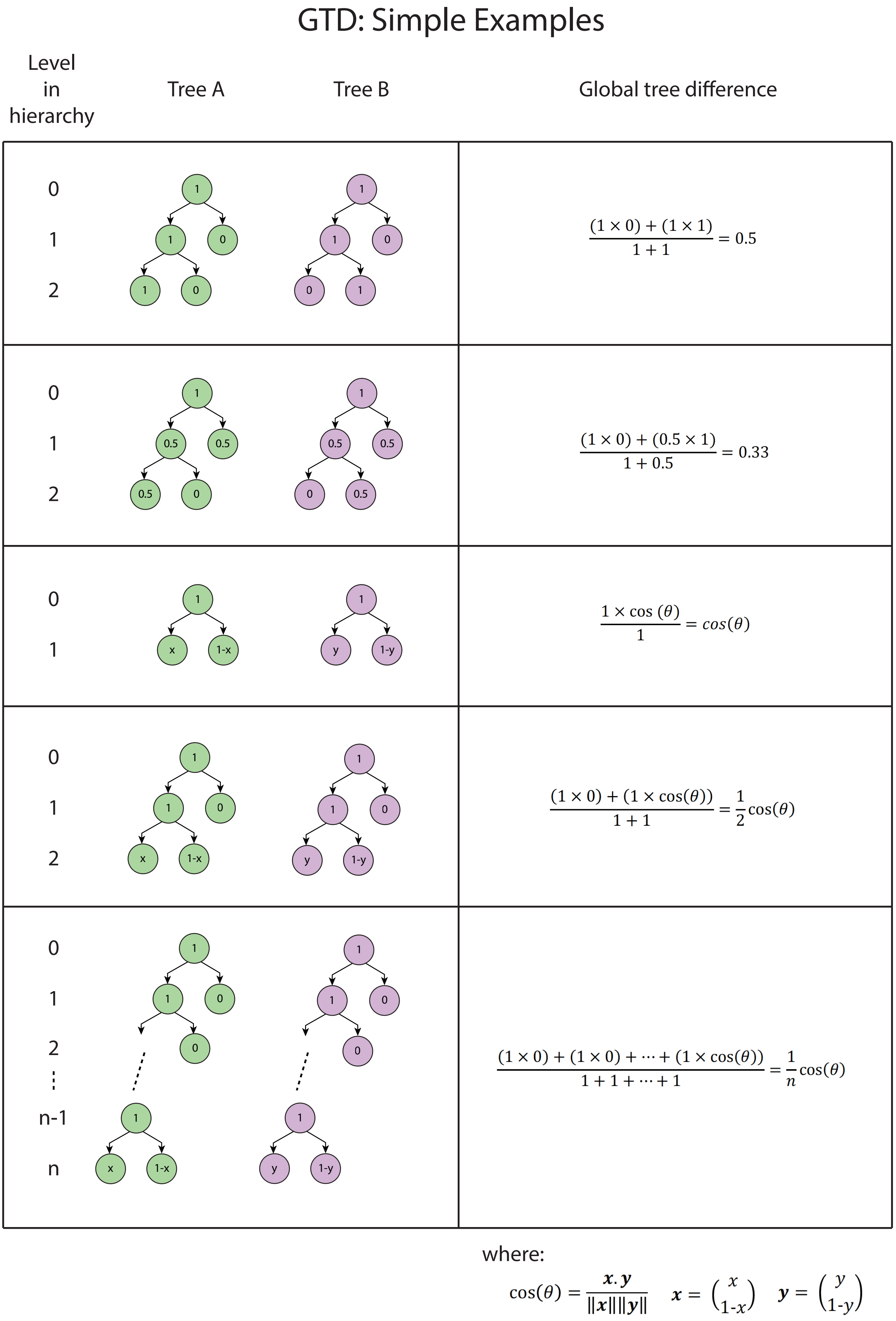

Supplementary Figure S2. Examples of simple trees and their global tree difference. Several examples of simple tree structure and their global tree difference values.

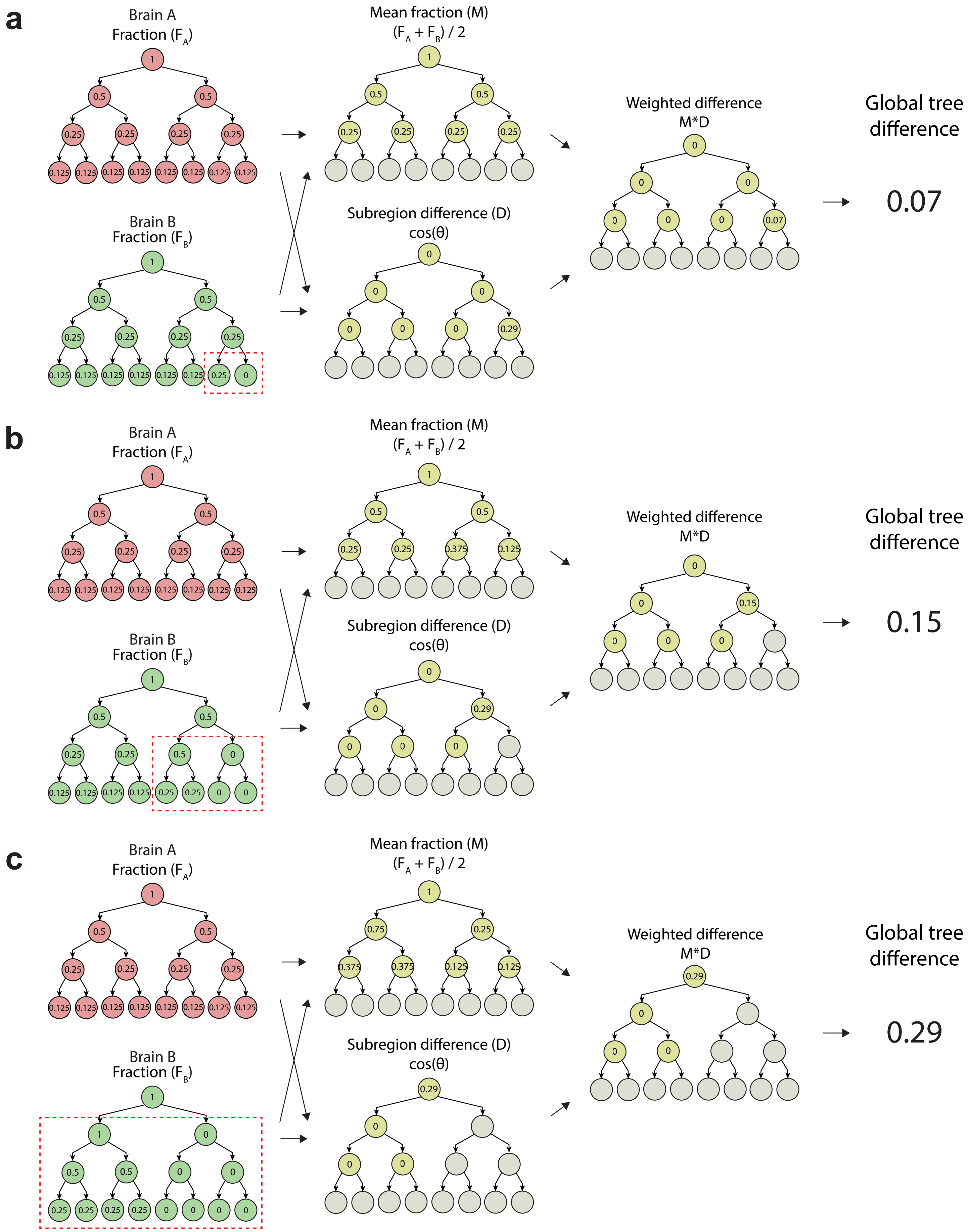

Supplementary Figure S3. More examples of trees and their global tree difference. More examples of trees and their global tree difference, highlighting the influence of hierarchy on the GTD value. In all cases (**a**-**c**), Brain A has cells distributed equally amongst its regions. In each case, Brain B has a single region in which cells are not distributed equally amongst its subregions. The effect on the GTD is dependent on where in the hierarchy that difference occurs. The GTD progressively increases as the difference is moved from low down (**a**), to the middle (**b**) and to the top (**c**) of the hierarchy. Red box highlights the parts of the tree which are different between Brain A and Brain B.

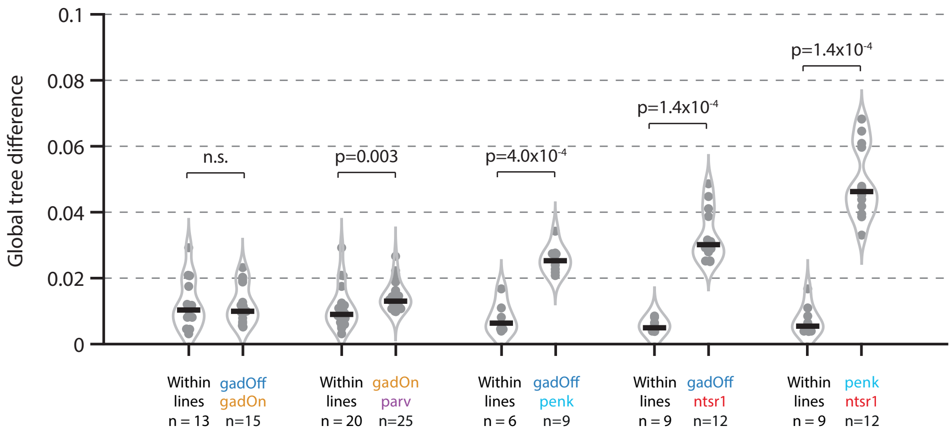

Supplementary Figure S4. Global tree difference showing similarity of input to L2/3 relative to L6.

Global tree difference for all pairs of brains between gadOff and gadOn experiments (left), between gadOn and parv, between gadOff and penk, between gadOff and ntsr1, and between penk and ntsr1 (right). For direct comparison, for each of these groups, the corresponding "within line" data is shown, where each brain in a mouse line was compared against the other brains in that line. (Related to Figure 1D).

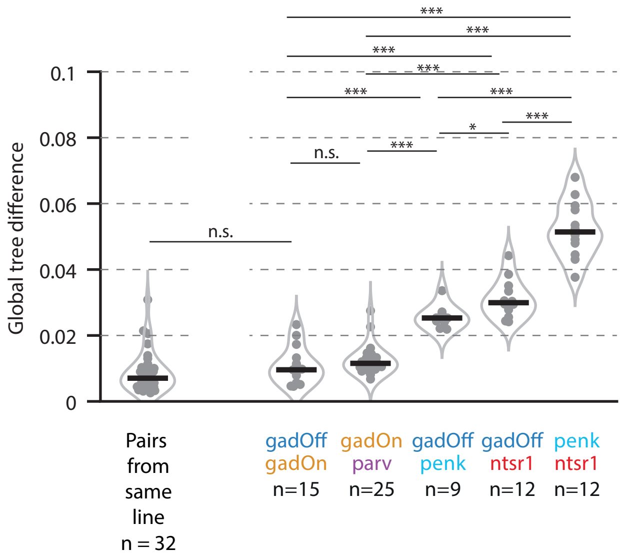

Supplementary Figure S5. Global tree difference recomputed with RSP removed from the ontology.
Global tree difference for each pair of brains from within the same line (left), and for pairs of brains between gadOff and gadOn experiments, between gadOn and parv, between gadOff and penk, between gadOff and ntsr1 and between gadOff and ntsr1 experiments (right). GTD value was computed with RSP removed from the ontology, and the difference between gadOff/gadOn and gadOn/parv is not significant. (Related to Figure 1D.)

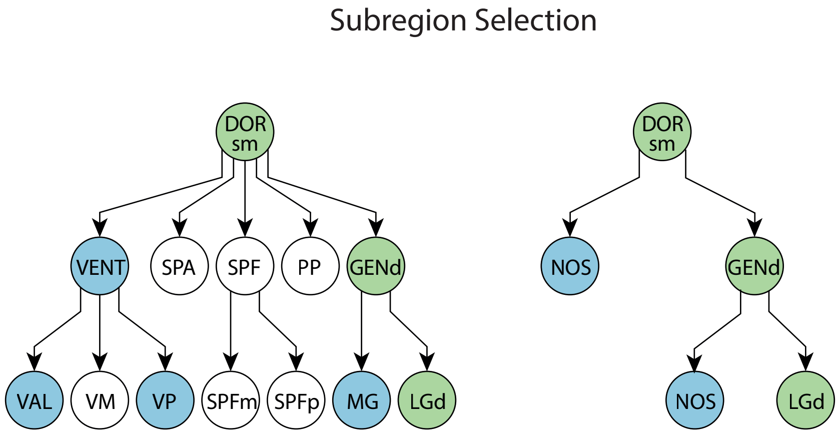

Supplementary Figure S6. Selection of regions for analysis.
An example of applying the subregion selection policy to the dataset. Left, a portion of the full ontology is shown from DORsm in the thalamus. Regions which meet the selection criteria (see Methods) are coloured in green. Regions containing some neurons but not meeting criteria for inclusion are shown in blue; regions with no neurons are shown as empty circles. Right, amalgamated ontology. Subregions not meeting criteria (blue) have been combined into a ‘not otherwise specified’ (NOS) subregion (which also included cells segmented to the parent but not any of its subregions). NOS subregions are included in the vector analysis stage, but are not further analysed.

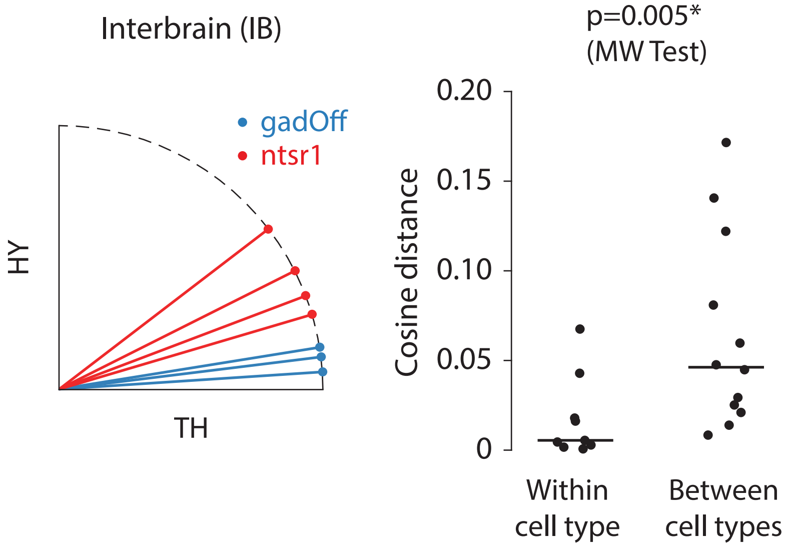

Supplementary Figure S7. Vector-space subregion analysis.

An example of the vector-space subregion analysis applied to the subregions of interbrain (composed of thalamus (TH) and hypothalamus (HY)). Left, the fractional composition of subregions for each experiment is plotted as a vector of unit length. Vectors are shown for the gadOff and ntsr1 groups. NOS fractions have not been shown, although they are always used in the analysis where present. Right, comparison of cosine distance between all pairs of vectors within mouse lines and between mouse lines.

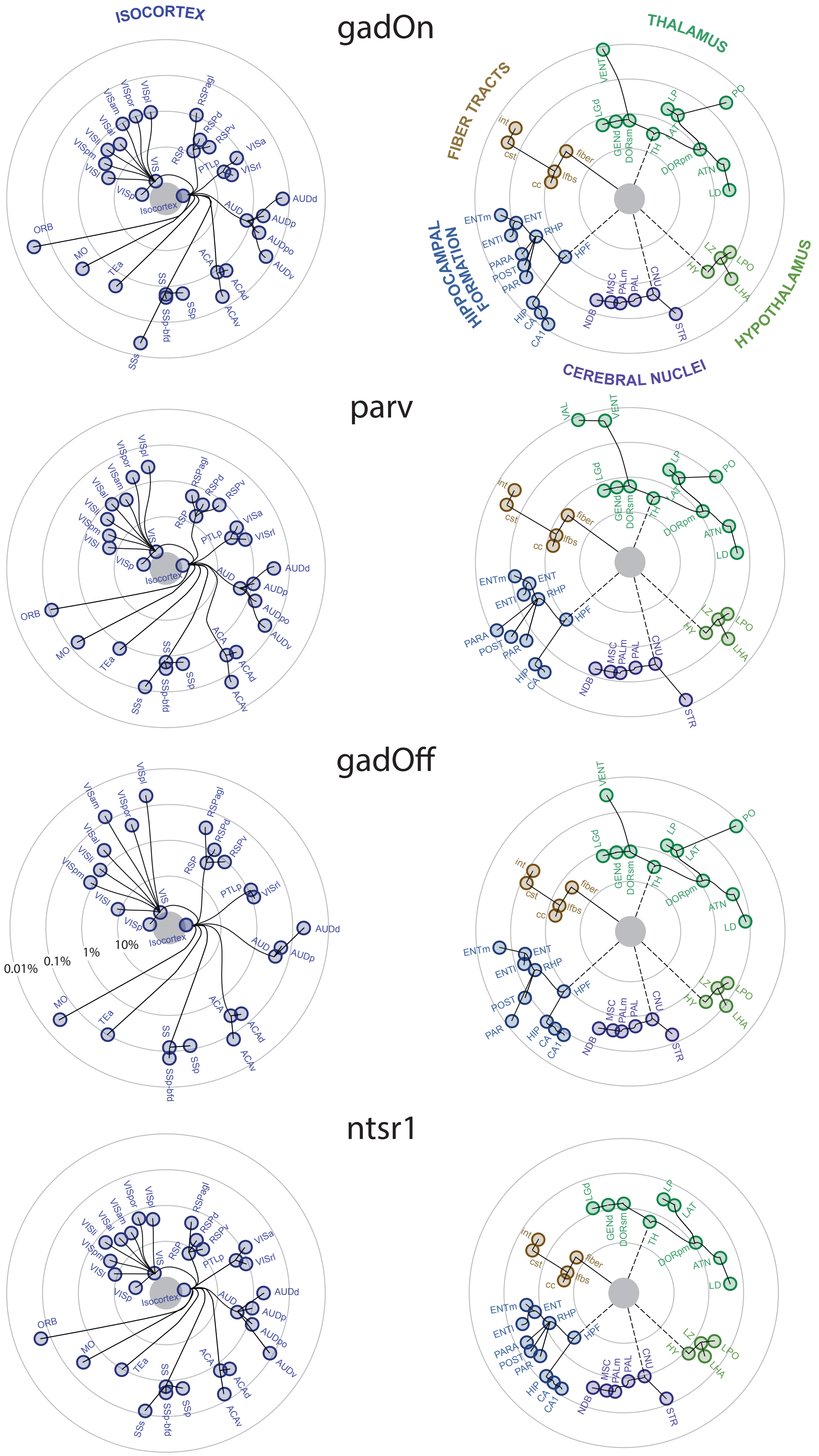

Supplementary Figure S8. Topography of presynaptic input to cell types of primary visual cortex. (Continued on next page.)

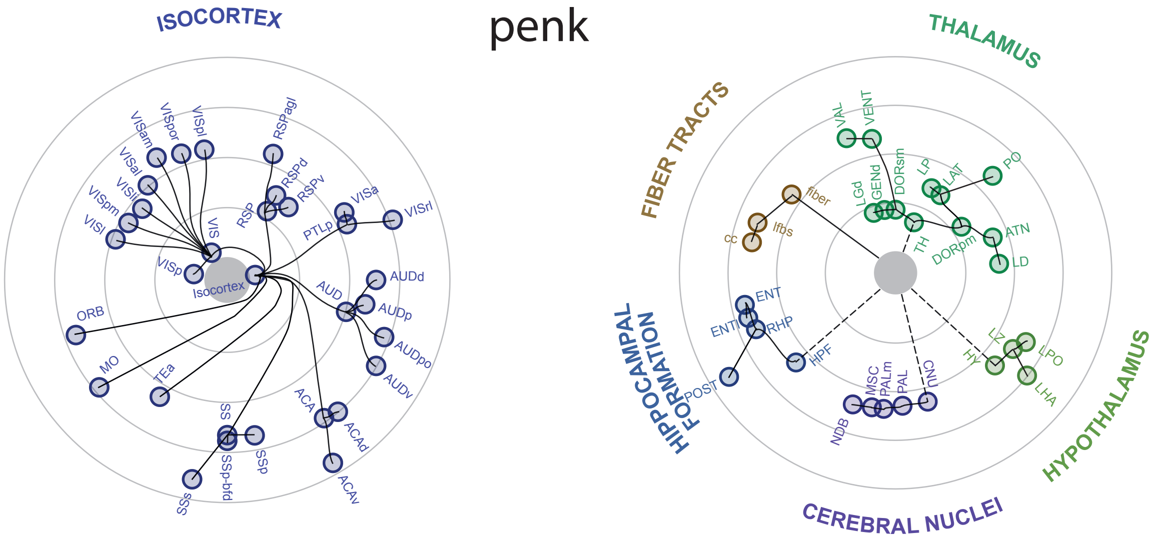

Supplementary Figure S8. Topography of presynaptic input to cell types of primary visual cortex. (Continued from previous page.)
Radial dendrograms showing the topography of average presynaptic connectivity to V1 for gadOn, parv, gadOff, ntsr1 and penk. Each node on the circular plot indicates a brain region, and the distance of the node from the edge of the centre circle indicates the average fraction of total rabies-infected cells found in that region. Lines connect brain regions appearing in the same branch of the Allen Reference Atlas ontology. Labels are consistent with the Allen Reference Atlas nomenclature.
